# Experiences of barriers to trans-sectoral treatment of patients with severe mental illness. A qualitative study.

**DOI:** 10.1186/s13033-020-00419-x

**Published:** 2020-11-30

**Authors:** Annette Sofie Davidsen, Johan Davidsen, Alexandra Brandt Ryborg Jønsson, Maria Haahr Nielsen, Pia Kürstein Kjellberg, Susanne Reventlow

**Affiliations:** 1 Department of Public Health, Section of General Practice and Research Unit for General Practice, University of Copenhagen, Oester Farimagsgade 5, 1014 Copenhagen K, Denmark; 2 Department of Health, VIVE–the Danish Center for Social Science Research, Herluf Trollesgade 11, 1052 Copenhagen K, Denmark

**Keywords:** Severe mental illness, Integrated care, General practice, Psychiatry, Social psychiatry, Recovery

## Abstract

**Background:**

Patients with severe mental illness (SMI) have shorter life expectancy than people without SMI, mainly due to overmortality from physical diseases. They are treated by professionals in three different health and social care sectors with sparse collaboration between them, hampering coherent treatment. Previous studies have shown difficulties involved in establishing such collaboration. As the preparatory phase of an intervention to improve physical health of people with SMI and increase collaboration across sector borders, we explored different actors’ experiences of barriers for collaboration.

**Method:**

We collected qualitative data from patients, professionals in general practice, psychiatry and social psychiatry involved in the treatment of these patients. Data consisted of notes from meetings and observations, interviews, focus groups and workshops. Analysis was by Interpretative Phenomenological Analysis.

**Results:**

The study revealed many obstacles to collaboration and coherent treatment, including the consultation structures in general practice, sectors being subject to different legislation, and incompatible IT systems. Professionals in general practice and social psychiatry felt that they were left with the responsibility for actions taken by hospital psychiatry without opportunity to discuss their concerns with psychiatrists. There were also cultural differences between health care and social psychiatry, expressed in ideology and language. Social psychiatry had an existential approach to recovery, whereas the views of health professionals were linked to symptom control and based on outcomes. Meanwhile, patients were left in limbo between these separate ideologies with no leadership in place to promote dialogue and integrate treatments between the sectors.

**Conclusion:**

Many obstacles to integrated trans-sectoral treatment of patients with SMI seem related to a lack of an overriding leadership and organizational support to establish collaboration and remove barriers related to legislation and IT. However, professional and ideological barriers also contribute. Psychiatry does not consider general practice to be part of the treatment team although general practitioners are left with responsibility for decisions taken in psychiatry; and different ideologies and treatment principles in psychiatry and municipal social psychiatry hamper the dialogue between them. There is a need to rethink the organization to avoid that the three sectors live autonomous lives with different cultures and lack of collaboration.

## Introduction

People with severe mental illness (SMI) often have comorbid physical diseases, and they are less likely to receive standard levels of care for most of these diseases [1]. Their life expectancy is 10–20 years shorter than for people without SMI [2], and most of the lost years are caused by death from physical diseases [3]. Several research projects have tried to unravel this problem and to find solutions [1, 4, 5]. Focus has been on collaborative work between medical and psychiatric care, either based in the hospital system [6–9] or involving the professionals from the primary health care sector [10–13]. Most of these programmes have shown disappointing results. Nevertheless, it has been mentioned that there is a great unrealized opportunity for developing new approaches that involve primary care [14], that it is important for patients with SMI to have access to primary care at their “reachable moment” [15], and that the general practitioner (GP) is responsible for both the treatment of the physical diseases and a structured care plan for these patients [16].

Patients with SMI, however, are often dependent on social care and support from the social sector to follow the treatment in general practice, and benefit from it [5, 17–20]. Nevertheless, research into collaboration involving social services is sparse and is mainly limited to reports from the UK [21, 22]. Studies describing interventions that use so-called ‘integrated care models’ have concerned hospital psychiatry and primary care.

Integrated care is an umbrella term comprising different models that focus on more coordinated and integrated forms of care provision [22–24]. A review of integrated care models [5] stated that few of the identified models were sufficiently described. The authors showed, however, that there was a need to empower both professionals and patients to remove the barriers to integrated care. They identified several areas that required special attention: improved communication between professionals and better IT solutions to support them; greater clarity about where the responsibility for the physical health care of this patient groups lies; and increased awareness of the effects of stigmatization where services are delivered [5]. A study of patient perspectives on integrated care explored how people with SMI and physical comorbidity manage their conditions, and that they viewed integration between primary and secondary care as important, not only in regard to the physical diseases. Continuity of care and listening skills from their primary care provider were also important in relation to the mental disease [10]. Another study showed that patients who received integrated care were more satisfied with their treatment than patients who received treatment separately from the primary and the secondary care sectors [7]. Two more studies concluded that integrated community care programmes with better medical treatment of concurrent chronic diseases might reduce physical disease and over-mortality, but that the effective ingredients in the mix were unknown [25], and that the implementation of different care models would require much effort and support [11].

Models of integrated care differ according to where they have been developed. Models developed in the hospital sector are often based on medical consultations in psychiatric clinics, or collaboration combined with active case finding and case management [9]. Models developed in primary care are often based on chronic care models and disease management, linked with behavioral interventions [24]. Patel and Chatterji argue that the most well-established delivery model is a team-based approach, with a case manager coordinating the collaboration between primary care and specialists. These authors state that there is sufficient evidence to implement approaches that integrate mental health care with treatment of physical diseases in primary care [26]. Many patients with SMI, however, need support from social services to take advantage of the different treatment offers in health care. Therefore, to be beneficial, an integrated care model should also involve social psychiatry [5, 17–20]. Consequently, we decided to make an intervention with a focus on collaboration between psychiatry, general practice and social psychiatry.

This study is part of a large-scale study (SOFIA) that aims to reduce excess mortality and improve need-related life quality among patients with SMI. In the present sub-study, the aim is to explore different professionals’ and patients’ experiences of trans-sectoral collaboration for patients with SMI and concurrent physical disease within the Danish health and social care system. This article will report findings from a two-year co-design approach [27] which formed the preparatory phase for a complex intervention in the SOFIA study. We explore the different sectors’ and some patients’ views on trans-sectoral collaboration for patients with SMI with the aim of revealing the barriers to collaboration and coherent treatment that must be taken into account.

## Method

The study was qualitative. A group of researchers collected data from patients and the different groups of professionals that would be involved in the subsequent intervention, in order to explore their needs and perspectives. The aim was to use these results to design a realistic and sustainable intervention that would be meaningful for and engage all of the actors [27].

### The Danish setting

In Denmark, patients with SMI receive treatment for their mental illness in hospital psychiatry, which includes hospital-based inpatient care and outpatient clinics. Patients in need of social support, for example supported housing or other social support, may receive this from what is termed “social psychiatry”. This is a department embedded in each of the 98 self-governing Danish municipalities, and their organizational structure may differ between those municipalities. Hospital psychiatry and general practice are embedded within each of the five Danish regions. The somatic diseases of patients with SMI are primarily treated in general practice, but in more severe cases patients may be referred to somatic hospitals. These are organized differently in the five regions, and are separate from hospital psychiatry. Chronic care belongs, primarily, in general practice. All treatment and social support is tax-financed and free for patients at the point of need.

General practice consists of independent, partly liberal, self-owned clinics which have a collective reimbursement agreement with the five Danish regions. General practice and hospital psychiatry are administered from separate departments in the regions. Social psychiatry is based in the municipalities and has psychiatric facilities for round-the-clock treatment, or support in the patients’ homes where patients are assessed for specific, well-defined forms of time-limited support. Some social psychiatric institutions are privately owned but paid for by the municipalities.

### Data material

The qualitative data for this study consisted of notes from different types of meetings, interview data, observation, focus groups, and workshops (Table 1).

**Table 1:** Types of data and participants

Types of data material	Participants
Meetings with social psychiatry and other professionals in six municipalities	Municipality 1: 2 leaders Municipality 2: leader plus 17 social workers Municipality 3: 2 leaders of social psychiatry; 3 psychiatric consultants Municipality 4: Leader of social psychiatry, leaders of 7 other municipal departments of health and social care Municipality 5: 3 leaders of different departments responsible for care of patients with SMI Municipality 5: leader of social psychiatry plus 2 leaders of sub-departments (biggest municipality)
Meetings with psychiatry in two regions	Region 1: Head of psychiatry Region 2: Head of psychiatry plus leader of main section
Observations at psychiatric facilities and social support in patients homes in two municipalities	Tree days in municipality 1 and 2 days in municipality 2
Interviews with three patients in social psychiatry Telephone interview with patient in user panel	2 men, 1 woman, age 46–56 years Woman in her twenties
Individual interview with social worker at a psychiatric facility	Woman, seniority 18 years
Individual interviews with two leaders of social psychiatry	Men, middle age
Two focus groups with general practitioners (GPs)	Group 1: 6 participants, 5 women, 1 man Group 2: 5 participants, 3 women, 2 men
Two trans-sectoral focus groups	10 participants in each group Group 1: 3 GPs, 1 M, 2 W 4 SPs, 1 M, 3 W 2 PSs, 1 M, 1 W 1 pharmacist, W Group 2: 2 GPs, 1 M, 1 W 5 SPs, all W 3 PSs, 1 M, 2 W
Two trans-sectoral workshops	20 and 7 participants respectively Workshop 1: 7 GPs 5 SPs, primarily leaders 4 PSs 2 practice staff 1 pharmacist 1 patient organisation leader Workshop 2: 4 SPs 3 PSs

### Data collection

Two of the five Danish regions were selected for the study. To gain initial information about the organisation of social psychiatry in the municipalities, we selected three municipalities in each of the two regions (out of 17 and 19 respectively). These municipalities differed in terms of the number of inhabitants (range 45.000-324.000) and the organisation of their social psychiatry services (based on information from their municipality websites). Meetings were established through contact with the head of social psychiatry, who invited professionals and sometimes other departments from the municipality to take part in them. As municipalities are broadly self-governed their organizational structures differ; their respective sizes also have an influence. This meant that in some municipalities the meetings mostly involved leaders, while in others professionals who had direct contact with citizens took part (Table 1). We took detailed notes during the meetings and expanded them afterwards.

Likewise, we established meetings with the head of psychiatry in the two regions to inform about the project and gain information about their attitude to collaboration. Notes were also taken from these meetings.

Based on contacts with social psychiatry, we established observations at two psychiatric facilities in two municipalities (5 days). A research assistant accompanied social workers in both inpatient psychiatric facilities and at visits in patients’ homes outside the facilities, took part in meetings about patients, and had conversations with patients. More formal interviews were made with three patients at a social psychiatric drop-in centre, with a social worker and with two leaders of social psychiatry in the two municipalities. In addition, a telephone interview was carried out with a patient from a user group.

Focus groups were held with GPs in the two regions, with five participants in one group and six in anothere. Two trans-sectoral focus groups were also held, one in each region with ten participants in each group and with participants from all three sectors: psychiatrists (PSs), GPs and professionals from the municipal social psychiatry (SPs) (Table 1).

Finally, two workshops were held. In one region this took the form of a bigger, day-long workshop with 20 participants coming from all three sectors, but it also involved practice staff from general practice and leaders from the municipalities and patient organisations. In the other region there was a half-day workshop with seven participants, psychiatrists and professionals from social psychiatry. This workshop took its point of departure in the problems defined in the first region. A detailed script with predefined steps and reflection cards was prepared in advance for the workshops, in order to guide the process towards concrete problem definitions and needs for change. The workshops were led by an anthropologist experienced in leading workshops, assisted by some of the authors. Data from the workshops took the form of detailed notes and accounts, as well as photos of display boards with drawings and post-it notes.

The interview and observation data were collected by three of the authors with expertise in different professional areas, e.g. a GP with psychiatric experience, an anthropologist, and a communication specialist. All individual interviews followed a semi-structured interview guide with open-ended questions. The interview guide was adapted to the specific groups of participants, and focused on the participant’s experiences in treating or having SMI or in caring for patients with SMI, and their experiences of the treatment system as well as of the collaboration between the different professionals and sectors involved in the treatment or care. For the focus groups there was a semi-structured guide which drew upon the participant’s hands-on experience in treatment of patients with SMI and cross-sectional collaboration. Observations followed an observation guide which focused on which problems the patients had, how they were dealt with and how the provision of cross-sectional care was enacted. The treatment of physical illnesses or comorbidities was also included in all interviews and observations.

All interviews and focus groups were audio-recorded and supplemented by field notes made by the attending researchers immediately after the sessions. All interviews and data from focus groups were transcribed verbatim by a student.

### Analysis

The detailed notes and the transcriptions of interviews and focus groups were analysed using Interpretative Phenomenological Analysis (IPA) [28].

The aim of IPA is to get close to the frame of reference of the person whose experience is being investigated and to understand the meanings that participants give to their experience. IPA developed from phenomenological psychology but has followed the movement of phenomenology in a more hermeneutic direction. There is an increased emphasis on interpretation, and the analysis is not exclusively based on description [29–31]. IPA is said to be “double hermeneutic”: the interviewer tries to make sense of the interviewee’s making sense of a phenomenon [32].

The first step of the analysis involved repeated readings of each transcript, or the notes from observations and meetings, to obtain an overall impression. The second stage involved marking text parts with keywords or phrases that reflected the meaning of the individual’s account. These phrases were developed into explorative comments focusing on core elements of each participant’s experience. The explorative comments were subsequently transformed into emergent preliminary themes for each participant. During this process the preliminary themes were gradually transformed into concepts representing a higher level of abstraction. We applied the same analytic process to each transcript, and a list of themes was compiled with extracts from each participant. To ensure the themes remained grounded in the data, the transcripts were re-read and marginal themes were excluded. Finally, higher-order “superordinate” themes were identified that represented participants’ perceptions and experiences across the material, and this formed the basis for the final write-up.

The superordinate themes identified from the data material were: system barriers for treatment in general practice; shared knowledge and collaboration; responsibility; cultural differences; and possible scenarios.

In the health care sector patients with SMI are called ‘patients’ but in the municipalities, and therefore also in social psychiatry, they are called ‘citizens’. To keep to a uniform terminology, we use the term ‘patient’ throughout the article, except in quotations.

## Results

Overall, the participants’ accounts revealed that patients with SMI were treated in three different, independent sectors: psychiatry, general practice and the municipalities. There was no overriding leadership for these different organizations and therefore no responsibility for, or guidance to, support the coherent treatment of patients. The three sectors lived rather autonomous lives with different cultures and with contacts between them largely dependent on individual professionals taking local initiatives to collaborate. Typically, the individual professionals felt that they were restrained from collaboration or met with a tangled bureaucratic system of indirect communication, with legislation in place to prevent direct contact.

Participants from general practice and from social psychiatry in particular considered trans-sectoral collaboration to be essential to improve the treatment of physical diseases in patients with SMI. These participants emphasized that more face-to-face meetings and better communication would enhance collaboration. Hospital psychiatry was less focused on collaboration, and did not express any need to collaborate with general practice.

We will describe the views of professionals from the different health and social care sectors below, and deal with aspects such as system barriers, lack of communication and sharing of knowledge between different organisations in health and social care, problems related to responsibility, and the influence of different treatment ideologies. Finally, we will describe some visionary views.

### System barriers for treatment in general practice

The participants agreed that the physical comorbidities of patients with SMI should be treated in general practice. However, professionals in social psychiatry said that the access to general practice and the reception therein was not well suited to these patients. It was difficult for patients to call and make an appointment, and it was difficult for them to sit in the waiting room. Their experience was that the consultations were too short for the patients to explain their symptoms, and they often did not feel taken seriously or treated equally.

The GPs said that their reimbursement system did not allow them to spend more than 15 minutes in consultations; patients with SMI did not fit into this current system of 15-minute consultations and later follow-up in new consultations. A GP said (in a non-judgmental way):

*They cannot find out how to behave as patients must do [to fit into the system], which means coming to the appointments and understanding the flow of the system. (GP, trans-sectoral focus group)*

#### Benefit of accompaniment

Both GPs and social workers viewed it as being helpful if social workers could support patients and accompany them to GP appointments. This would reduce non-attendance rates and give patients support in bringing forth their complaints as well as remembering the different topics. Furthermore, the agreed treatment or plan would be shared with the social worker, which would support patients with cognitive problems:

*Many GPs appreciate that a social worker calls together with the citizen and/or comes together with the citizen, in that it may prevent some misunderstandings. It can also contribute to the citizen actually turning up. It is difficult for many citizens to arrive when they have an appointment. (Social worker, interview)*

*If the social worker and psychiatric facilities could in any way get more resources to support this, or at least get them [the citizens] to the GP. It is often difficult for them [the patients] to get going. (GP, trans-sectoral focus group)*

Patients also appreciated being accompanied to appointments because they often lost concentration; it could then be difficult to explain one’s symptoms and understand what was said in response. It was often not possible, however, for social workers to field messages for patients or accompany them to the GP, because these tasks had to be agreed upon administratively as part of the patient’s care plan in the municipality.

### Shared knowledge and collaboration

Patients, GPs, and social psychiatry expressed a common desire that the different professionals working with a patient should have access to and the ability to update the same knowledge. This desire, however, met several obstacles. For some this was due to a lack of awareness, especially by psychiatry, of the other sectors’ need for information; for others it was caused by legal issues and incompatible IT systems across sectors.

#### Information to GPs from psychiatry

GPs said that, contrary to somatic health care, they did not receive information from psychiatry during the long periods of patients’ outpatient psychiatric treatment. Neither did they always receive discharge letters following a patient’s discharge from a psychiatric hospital. Often GPs did not know whether or not their patients were still being treated by psychiatry:

*I think it is really difficult to collaborate with outpatient psychiatry because we hear nothing from them. It is actually FMK [electronically shared prescription record] that is the lifeline, so you could say: “Okay, they are seen there, because some medicine has been prescribed”, but you have no contact with them. Not even when they are discharged, so it is a bit uncertain. (GP, focus group with GPs)*

*The discharge letter is not only missing on discharge. It is also missing during the ongoing psychiatric course [of outpatient treatment]. Sometimes you have no idea if they are still in outpatient psychiatry. (GP, focus group with GPs)*

If GPs tried to contact psychiatry by telephone, they said that they often got through to a psychiatrist who did not know the patient, and not the psychiatrist or nurse involved in the patient’s treatment.

*It is really difficult for us to get into contact with psychiatry. We lack a system that, without too much effort, you could speak with the doctor who is responsible for the treatment and who knows the patient. (GP, focus group with GPs)*

Hospital psychiatry did not express any lack of, or need for, further cooperation. They saw themselves as offering their expert knowledge and support to professionals from the other sectors. Psychiatry leaders said that they could not offer any structured support to general practice as it would not fit into their working routines. They imagined that the collaboration would demand a special staffing for this task and did not view collaboration as an integrated part of their work. Accordingly, they referred to a lack of resources as the reason for the lack of collaboration:

*It is a question of resources. What you ask for is increased access. Having a way of being more accessible to each other, that would be very desirable. We also want that but we just do not have the resources for it. (Psychiatrist, trans-sectoral focus group)*

A few consultants in local outpatient psychiatry, however, said that they were open to telephone contact to give their expert advice to GPs but did not express any need for mutual collaboration.

#### Collaboration with social psychiatry

##### GPs’ perspectives

GPs said that they were unable to communicate directly with social psychiatry and described two different kinds of problem: first, the two sectors are subject to different legislation (The Consolidation Act on Social Services and The Health Act), and professionals are not allowed to share information across sector domains without written, informed consent from the patient. Second, although patients were generally willing to sign this consent, the two sectors used different and incompatible IT-systems which made it difficult to communicate about the patient’s records.

GPs said that it could be very difficult to contact social psychiatry directly, which was a frustrating barrier:

*I have to call this big centre, where they answer the phone between 9 AM and a quarter past nine or something like that, and talk with someone who will then pass on the message to someone else. And that works really, really poorly. Then I have been told recently that we have to write to the municipality. Then I fill in a social medical certificate and make them aware of the problem and ask them to contact the patient. But it would be nicer to have a face-to-face [meeting] or at least the phone and talk together instead. (GP, focus group with GPs)*

##### Social psychiatry’s perspectives

Beyond the problems regarding the individual patient consultations, social psychiatry described diverse experiences of collaboration with GPs. At an overriding level, some of the leaders expressed that they experienced a lack of commitment to collaboration from GPs. They interpreted this as being based on different professional cultures:

*There is a general experience of imbalance as regards the GPs’ commitment to collaboration. Generally, they are not as committed in their collaboration as we are. There are differences in both traditions and culture. (Leader, social psychiatry)*

At the level of cooperation between social workers and individual GPs, the experiences differed a lot. Some social workers had experienced unfriendly communication, and some reported that they had even been reprimanded by a GP if they phoned about a patient. Other social workers said that if they wanted to phone a GP on behalf of a patient, they had to do this on equal terms with other patients and not as professional collaborators. This meant taking their place in the telephone queue in the morning (8–9 AM). These experiences made them think that they were not acknowledged as professional collaborators. However, other social workers praised their collaboration with cooperative GPs.

Professionals from social psychiatry also described experiences of a lack of cooperation between somatic and psychiatric hospital departments. A social worker told of a young woman who had serious cardiac complications from psychopharmacology. However, the psychiatrist refused to follow the advice from the cardiologist, saying that a reduction in psychopharmacological medication could worsen the patient’s mental state. The social worker questioned why psychiatry and somatic health care were not more interested in collaboration:

*I wonder why you would not have a professionalism and a curiosity about collaboration—that you do not trust each other’s professional competences a little more. (Social worker, interview)*

There was no direct contact between the psychiatrist and the cardiologist, and the social worker had to be both the messenger and the negotiator.

##### Psychiatrists’ perspectives

Psychiatrists complained about accessibility and bureaucracy in the municipalities, which hampered the possibility of their getting support from social psychiatry.

*All that bureaucracy, if you could remove that, then I think that things would go more smoothly….it takes ages just to find out if you can get in contact. (Psychiatrist, trans-sectoral focus group)*

##### Patents’ perspectives

All interviewed patients said that they experienced no collaboration between the different professionals in the three sectors: “There is no collaboration at all. There is [really] no collaboration at all.” (Patient 1).

The patients viewed their social worker as the most important person for their treatment, for keeping track of the different treatment modalities and as a sort of advocate to be accompanied by in heath care appointments. This was especially the case in psychiatry appointments, where they often did not feel heard:

*I have never ever felt that I have been to a psychiatrist where it was positive. I think it is so much focused on medication instead of actually talking to a person about how he is doing. That’s how I have felt. And the physical, the psychiatrist did not care about that (Patient 3)*

However, patients said in interviews that this was not always possible, because social psychiatry was dominated by what the social workers were *not* allowed to do and that there were only a few tasks which they *were* allowed to do.

*I sometimes think that the things they are not allowed to do puts a brake on municipal services. It’s an area exposed to many savings. There are not many tasks they can take on (Patient 4)*

The patients we interviewed were satisfied with the GPs they had now, and sometimes actually felt that the GP would side with them against psychiatry. They felt they were taken seriously and listened to by the GP. They also, however, tried to be accompanied by their social worker to their GP if they feared not getting their message across.

The patients we interviewed had, however, also experienced GPs that were “incredibly superficial” and had then chosen another GP, often in single-handed practice to secure continuity: “Well, it was just so in and out, then it is ‘bong’ finished. But he [the current GP] is really good.” (Patient 2)

### Responsibility

GPs and social psychiatry considered that there was a problem of responsibility. Often it was not clear who had the responsibility for the patients’ overall treatment. In the social psychiatric facilities, the social psychiatry professionals said that “the municipality and the regions fight over who has the responsibility”. This was in reference to when patients were too ill to be kept in the community facility and needed to be readmitted to hospital (or not dismissed yet), and it also concerned more cultural differences as described below.

The GPs considered the question of responsibility as problematic. They were often left with the task of prescribing psychopharmacological medication to their patients - medication which they had not themselves given an indication for or started. They feared that if they did not follow the rules of control, ordering blood tests or ECGs for patients taking specific medications, for example, the matter could be referred to the complaints board. They also had to assess the possible interactions of psychopharmacological medication with the somatic medication they wanted to prescribe, but GPs were not always informed of the psychiatric medication their patients were taking.

*There is much information about what the doctor ought to do—which means should* *do. As to QTc [electric disturbance in the ECG], whose responsibility is it if something happens to them [the patients]? I think it is difficult to bring outpatient psychiatry in to take responsibility for this and get them to say: “Together with what you [the GP] do, this is all right.” They don’t [say that] (GP, focus group with GPs)*

*Who is actually responsible? Something about responsibility, that is a mess. And you do not know what each other is doing. (GP, trans-sectoral focus group)*

In principle, each doctor who prescribes medication for a patient has the responsibility for overseeing all of the medication, which the GPs considered a frightening challenge. If patients were dismissed from psychiatry, often because they did not fit into any specialized department or package, or due to non-attendance, it was the GP’s responsibility to continue the medication. This was often a long list, with no opportunity to contest it with psychiatry.

Social psychiatry agreed on these challenges:

*Communication between the different [sectors] about these citizens. There we are challenged. Who takes the responsibility for the long medication list, where many providers have prescribed something? The interaction between treatment psychiatry and psychiatric facilities, or social workers and GPs? Perhaps it is about having some formalized collaboration agreements (Leader, social psychiatry)*

### Cultural differences

Health and social care professionals described their interventions based on different theories and ways of thinking. The participants from health care focused on the treatment of diseases, and both general practice and psychiatry expressed a wish for social psychiatry to upgrade their health professional competences to better support the health of their patients. Participants from social psychiatry, however, built their interventions on social pedagogic thinking involving a focus on recovery, the autonomy of the individual, and social pedagogic methods. They did not consider health issues as their primary task.

*How will it then play with our social pedagogic methods, because they are our primary tasks? There will be some resistance to it, because, again, it is a health professional thing, which actually is not our primary task (Social worker, trans-sectoral focus group)*

Professionals from social psychiatry described their different approaches using concepts such as *goal ladders* and metaphors like *strength flowers*. They used different abbreviations for their theoretical approaches, which, although deriving from pedagogic theory, were not the same in the different municipalities. At leader level in particular, professionals from social psychiatry used quite another language than the language of health care professionals. They talked about “discovering yourself”, how exercise, for example, “could lead to a psychological and social dimension that could overcome the health condition,” and how this should be integrated into the recovery process.

Social psychiatry leaders said that although the language and thinking differed between the health and social sectors, these differences could be overcome by meeting face-to-face and gaining insight into each other’s tasks and working methods.

*Our way of thinking—there are some cultural differences, which you must work on. Many of these preunderstandings of each other we could lay down by actually meeting, meeting physically, getting to know each other and getting some insights into each other’s tasks and ways of solving the tasks, which would open things up for some other understandings. (Leader, social psychiatry)*

Each sector had its own focus and perspectives, which did not necessarily involve collaboration with the other sectors. This was especially the case for psychiatry, where professionals responded by trying to incorporate an element of giving expert advice into their regime. Moreover, regional psychiatry programmes did not mention general practice or the municipalities as parties involved in the patients’ treatment.

### Possible scenarios

Some participants from the municipalities and some GPs described their visions of possible scenarios for a collaborative system, some of which were deemed possible if all actors could agree on them. These participants said that a team-based organization with collaboration between all three sectors would be meaningful and that collaboration had to be based on face-to-face meetings. Leaders from social psychiatry said that a real collaboration between regional psychiatry and municipal psychiatric facilities would help the facilities to work with patients in a more qualified way. This would prevent hospital re-admissions, often caused by lack of psychiatric support, particularly in terms of medication:

*Patients’ treatment is not finished at [discharge from] the hospital. Then there are more re-admissions. And the psychiatric facilities are not capable of dealing sufficiently with the target group. Perhaps you could prevent re-admissions through collaboration. If they [the patients] are going to have a new medication or if a psychiatric treatment needs to be adjusted, then there should, of course, be a close collaboration. I actually consider that this should be handled by the [municipal] facilities. They must then, to a necessary degree, be supported by nurses or doctors. But in my view, this is a municipal task, and they must be able to deal with these citizens (Leader, social psychiatry)*

In line with this view of giving social psychiatry increased power to relieve the pressure on psychiatry, GPs also mentioned that some patients could be handed over to GPs in a shared care model. This would, again, relieve psychiatry, which described its problems as a lack of resources:

*I think, if they are so busy in outpatient psychiatry, then they should perhaps let go of some of the patients, and then you could make shared care. I know that you should not come up with this [idea] in these times but if we more or less [have the patients] anyway? (GP, trans-sectoral focus group)*

Psychiatry did not, however, see how these proposals could offer relief but rather considered it as a new burden for them.

The social psychiatry leader quoted above also reflected on the whole organizational set-up for patients with SMI. He described the process of de-institutionalization which began in the 1980s. This had, in his opinion, led to a different form of institution in the shape of these often large municipal psychiatric facilities, which did not live up to the thinking behind de-institutionalization:

*We have talked of de-institutionalizing since the ’80s, and now they are sitting at these psychiatric facilities, but is that an inclusive way of living a life? Or is it a mirage we have developed? Are they de-institutionalized? Are they integrated, are they part of the community? We have closed the institutions, but we have just created some decentralized, closed units around the country, which we do not have a snowball’s chance of controlling because most of them are private. And without ceremony, we pay 700.000 to 1.000.000 DKK per year for having a citizen there (Leader, social psychiatry)*

This leader advocated for greater activation of civil society with, for example, peer-workers and a focus on reducing psychopharmacological medication. This could, however, not be achieved for citizens who had become chronic patients in the system:

*Now they have been placed—750 citizens in six municipalities in this way. They are so badly off and their medication makes them even worse, so they cannot be moved out—a Gordian knot (Leader, social psychiatry)*

Even for these patients, however, trans-sectoral teams involving municipalities, GPs, and psychiatry with outreach capabilities, were considered the best solution.

Some particularly engaged GPs advocated for leadership of new initiatives and felt that GPs were in a strong position to take the lead in this process. One participant described local initiatives driven by committed professionals, which had achieved good results:

*Much of it is leadership. That you sit down in municipal psychiatry and talk with regional psychiatry and talk with the KLU [committee with members from general practice and the municipality] about getting coherence in your municipality and say “what can we do that makes sense and which we all think we can work with.” You have to sit down and ask “what is it that we want to do together all of us?” We actually tried it with the psychiatric facilities and municipal psychiatry. And they were so glad that we came. They thought we were not interested, but we were interested. We also sit with the problem (GP, trans-sectoral focus group)*

## Discussion

Our findings showed that there were many obstacles to coherent treatment of patients with SMI. These comprised organizational elements, legal issues, IT-system incompatibilities, cultural differences and differences in theoretical approaches, and no overriding leadership. The consultation structure in general practice was not considered suitable for patients with SMI. Information was not shared between the different sectors, partly due to a lack of awareness from professionals in hospital psychiatry that collaboration was necessary. The lack of clarity around responsibility was a big challenge, especially for GPs, who often feared litigation by “Styrelsen for Patientsikkerhed” (The Danish Patient Safety Authority). GPs felt that they were left with overall responsibility for their patients, including for medication which they were not familiar with. Social psychiatry experienced the same lack of clarity around responsibility when patients were discharged from hospital psychiatry into municipal psychiatric facilities. Patients considered the social worker in social psychiatry as the most important person for their treatment and actually an advocate to secure their being heard especially in psychiatry.

Legal challenges were especially noted in relation to social psychiatry, which belonged to a different branch of legislation than health care. Communication about patients is, therefore, forbidden without their written, informed consent. In addition, social psychiatry used a different IT-system which was not compatible with the system in health care. The social pedagogic language and approach in social psychiatry differed from the thinking in health care, and without face-to-face meetings to promote learning about each other’s tasks and working methods, cultural differences were upheld.

The challenges experienced by the participants in this study are in line with those shown in the literature: treatment is fragmented [8], collaboration is sparse, and an integrated care approach requires much effort and support [11]. Authors have shown that there is a need to empower professionals to remove barriers and improve communication between them, but that there is also a need for IT solutions to support communication and for clarity on responsibility [5]. None of these needs were fulfilled in the present study where professionals were left on their own without any overriding, empowering leadership, and with both legal and technical barriers for communication between sectors.

Literature has shown that patients want integrated care and shared knowledge [7]; that they view general practice as important for continuity of care, and that they value GPs’ listening skills [10]. This corresponds to the findings in the present study, where collaboration was hampered by the lack of information flowing from psychiatry to general practice. Psychiatry seemed to be very closed off from the other sectors and did not consider the GPs or social psychiatry to be part of the treatment team. Patients also experienced this lack of cooperation and the need of support from the social worker in their meetings with psychiatry.

The consultation structure in general practice, and the obstacles for social psychiatric support in these consultations was also a hindrance to collaboration. The consultation system in general practice, supported by the collective agreement with the regions [33], did not suit patients with SMI. Due to emotional, social, and cognitive problems they need a different form of consultation than the standard 10-15 minute model [34]. In the present study, professionals from social psychiatry pointed out that access to and reception in general practice did not match the needs of these patients. Poor accessibility, perhaps combined with a poor attitude by GPs and too little time in the consultation, have been shown to negatively influence health outcomes [35].

Different integrated care models have been described in the literature [3, 24, 26]. The participants in the present study, who had some visionary ideas, proposed team-based integrated care models with participation from all three sectors and with less rigidity, especially from psychiatry. It was also suggested that psychiatry should take part in more outreach activities by offering professional support to municipal psychiatric facilities. GPs proposed that more patients should be discharged to general practice from outpatient psychiatry and included in a shared care model. Psychiatry, however, did not buy into these proposals.

Shared care models have been tested for patients with different kinds of mental disease, but results show that much support is needed to make them work [11]. A Cochrane review studying collaborative care between psychiatry and general practice for patients with SMI only succeeded in including one study—it did, nonetheless, show improved quality of life for patients [12]. Studies involving all three sectors are nevertheless lacking. Community mental health teams (CMHTs) in the UK may involve elements of collaboration that also include the social sector. A Cochrane review about CMHTs found insignificant results, although death was consistently lower in the CMHT group [36].

Barriers to integrated care are found on many levels, from clinical practice to administration and regulation [3, 37] but integrated care models are also dependent on having an overriding leadership [21]. In the present study there was no administrative or organizational support in place to establish collaboration between the sectors and the professionals, and there was no overriding leadership.

The study also showed that the health and social sectors were governed by different ideologies. Social psychiatry acted from a recovery perspective, whereas medicine, especially psychiatry, seemed to focus on outcomes and medication. GPs had a more generalist approach but still felt an obligation to continue the medication prescribed by psychiatry. The concept of recovery has a long history, originally defined by clinicians and based on medical outcomes [38]. During the 1960s and 1970s, the consumer movement gave rise to a more process-oriented view of recovery [39]. Anthony [40] described recovery existentially, as finding new meaning and purpose in life after the experience of mental illness. The two meanings of the concept led to a polarization in the understanding of recovery. It has since been shown, however, that this polarized view may be unfounded [41] and the two meanings have eventually started to become united in more recent research. Studies of patients’ views on recovery have shown that symptom relief, better cognitive function, improved social functioning, and participation in community life are important [39, 41–43]. Patients consider that factors such as the ability to control symptoms, finding the right kinds of medication, and their relationship with the psychiatrist, are also important for recovery [42].

In the present study, only social psychiatry talked about recovery, which was seen as the basis of their social pedagogic approach. Their view seemed, however, only to cover the existential definition of recovery where patients should “discover themselves” and “the psychological and social dimension should overcome the health condition”. The health professionals did not talk about recovery but rather symptom control and the steering of medication, which can be seen as the other end of the polarized understanding of recovery based on outcomes. If patients are not to be left in limbo in this polarized view, where the help they get is based on two separate ideologies, these views have to be united [41, 42].

### Limitations

We contacted psychiatry in two of the five Danish regions and six out of 36 municipalities. The 98 Danish municipalities are self-governing and although we sought to choose the municipalities to obtain maximum variation, we cannot be sure that these municipalities cover the views of all municipalities. Likewise, the number of professionals in interviews, focus groups and workshops was relatively small. However, we recruited different participants to the different forms of data collection, and different researchers took part in the data collection. Nevertheless, the informants gave corresponding accounts across regions and municipalities, and the problems and needs that emerged in the different forms of data were the same. We think, therefore, that the information can be transferred to other regions, municipalities and general practices in Denmark and perhaps to other similar health and social care systems. We made observations in two municipalities, including observation at psychiatric facilities and in patients’ homes and carried out interviews with patients there. The number of patients interviewed was rather small, and possibly patients willing to be interviewed were the most well-recourced. They had all, in particular, been able to select a GP with whom they communicated well. Nevertheless, their views were rather similar, especially as regards their dependence on their social worker and their attitude towards psychiatry, which means that their experiences are probably shared by other psychiatric patients. However, patients’ perspectives are studied in further detail in a sub-study with specific focus on patients and will be reported elsewhere.

## Conclusion

Integration of the different views and treatments would demand an overriding leadership to promote dialogue between the sectors, encourage initiative, and integrate coherent treatment. There is no such initiative forthcoming from the leaders of regions, the government, hospital psychiatry, or from the organization of GPs. In the present study, some GPs and social psychiatry leaders advocated for committed professionals to start the process from the bottom. They proposed that local GPs should take the initiative in the municipality where they worked, and social psychiatry leaders proposed a greater focus on civil society and the involvement of the community, for example by using peer-workers.

Although such bottom-up initiatives can be valuable, they cannot solve the problem of an incoherent system characterized by a lack of collaboration, unclear points about responsibility, legal and IT challenges, different cultures, ideologies, and views on the treatment of patients with SMI. All these challenges lead to patients often experiencing problems in the system and gaps and inconsistencies in their treatment. There seems to be a need to rethink the whole organization and to integrate views from all sectors in a collaborative, team-based effort that includes specialist psychiatry, general health care, and social pedagogic perspectives in the holistic treatment of patients. In addition, there is a need for dialogue between the proponents of the two polarized views of recovery to agree on a more balanced and united view as a possible condition for integration of regional and social psychiatry and shared care with general practice, which could even relieve the pressure on psychiatry. Both general practice and social psychiatry seemed willing to do this, if there were opportunities to spar with a psychiatrist on patient treatment, to share information as with somatic medicine, and to restore outreach activities between psychiatry and social psychiatry and psychiatric facilities in the community. The resources to provide these services are lacking in today’s regional psychiatry model, and further research is required to see if the investment would be effective.

This study forms the preparatory phase for an intervention to improve the somatic health of patients with SMI which will have its point of departure in general practice. The conditions in psychiatry, the IT and legislative barriers, and municipal bureaucracy will also exist in the intervention. We will take the findings of the present study, however, and test whether educational elements and knowledge sharing with professionals in the different sectors will increase collaboration.

## Data Availability

The participants were assured that their that their confidentiality and anonymity would be protected. Therefore, the research data is not available publicly because this would compromise this promise, participants’ privacy and our ethical approval.
